# Multi-omics subgroups associated with glycaemic deterioration in type 2 diabetes: an IMI-RHAPSODY Study

**DOI:** 10.3389/fendo.2024.1350796

**Published:** 2024-03-06

**Authors:** Shiying Li, Iulian Dragan, Van Du T. Tran, Chun Ho Fung, Dmitry Kuznetsov, Michael K. Hansen, Joline W. J. Beulens, Leen M. ‘t Hart, Roderick C. Slieker, Louise A. Donnelly, Mathias J. Gerl, Christian Klose, Florence Mehl, Kai Simons, Petra J. M. Elders, Ewan R. Pearson, Guy A. Rutter, Mark Ibberson

**Affiliations:** ^1^ Centre de Recherche du CHUM, Faculty of Medicine, University of Montreal, Montreal, QC, Canada; ^2^ Vital-IT Group, SIB Swiss Institute of Bioinformatics, Lausanne, Switzerland; ^3^ Section of Cell Biology and Functional Genomics, Department of Metabolism, Diabetes and Reproduction, Imperial College of London, London, United Kingdom; ^4^ Janssen Research and Development, Philadelphia, PA, United States; ^5^ Department of Epidemiology and Data Sciences, Amsterdam University Medical Center, Amsterdam, Netherlands; ^6^ Amsterdam Public Health, Amsterdam, Netherlands; ^7^ Department of Cell and Chemical Biology, Leiden University Medical Center, Leiden, Netherlands; ^8^ Department of Biomedical Data Sciences, Section Molecular Epidemiology, Leiden University Medical Center, Leiden, Netherlands; ^9^ Division of Population Health & Genomics, School of Medicine, University of Dundee, Dundee, United Kingdom; ^10^ Lipotype GmbH, Dresden, Germany; ^11^ Department of General Practice and Elderly Care Medicine, Amsterdam Public Health Research Institute, Amsterdam UMC–location VUmc, Amsterdam, Netherlands; ^12^ Lee Kong Chian School of Medicine, Nan Yang Technological University, Singapore, Singapore

**Keywords:** multi-omics, type 2 diabetes, glycaemic deterioration, metabolic syndrome, lipidomics, proteomics

## Abstract

**Introduction:**

Type 2 diabetes (T2D) onset, progression and outcomes differ substantially between individuals. Multi-omics analyses may allow a deeper understanding of these differences and ultimately facilitate personalised treatments. Here, in an unsupervised “bottom-up” approach, we attempt to group T2D patients based solely on -omics data generated from plasma.

**Methods:**

Circulating plasma lipidomic and proteomic data from two independent clinical cohorts, Hoorn Diabetes Care System (DCS) and Genetics of Diabetes Audit and Research in Tayside Scotland (GoDARTS), were analysed using Similarity Network Fusion. The resulting patient network was analysed with Logistic and Cox regression modelling to explore relationships between plasma -omic profiles and clinical characteristics.

**Results:**

From a total of 1,134 subjects in the two cohorts, levels of 180 circulating plasma lipids and 1195 proteins were used to separate patients into two subgroups. These differed in terms of glycaemic deterioration (Hazard Ratio=0.56;0.73), insulin sensitivity and secretion (C-peptide, *p*=3.7e-11;2.5e-06, DCS and GoDARTS, respectively; Homeostatic model assessment 2 (HOMA2)-B; -IR; -S, p=0.0008;4.2e-11;1.1e-09, only in DCS). The main molecular signatures separating the two groups included triacylglycerols, sphingomyelin, testican-1 and interleukin 18 receptor.

**Conclusions:**

Using an unsupervised network-based fusion method on plasma lipidomics and proteomics data from two independent cohorts, we were able to identify two subgroups of T2D patients differing in terms of disease severity. The molecular signatures identified within these subgroups provide insights into disease mechanisms and possibly new prognostic markers for T2D.

## 1 Introduction

It is increasingly recognised that type 2 diabetes (T2D) presentation differs markedly between patients (1). Nevertheless, approaches using current clinical measurements and classical laboratory markers are likely to be underpowered to capture the underlying heterogeneity within the disease (2). Better characterization of T2D at the molecular level is likely to be essential to move from a “one size fits all” approach to more precise health management of this disease (3).

Compared to traditional approaches, unbiased “multi-omics” analysis could potentially identify reliable patient-specific biomarkers for disease progression. Previously, using three T2D cohorts, the Hoorn Diabetes Care System (DCS), Genetics of Diabetes Audit and Research in Tayside Scotland (GoDARTS) and All New Diabetics in Scania (ANDiS), encompassing 2,973 individuals across three molecular classes, metabolites, lipids, and proteins, we were able to identify several novel biomarkers for T2D progression and prevalence (4). Based on both Cox and Logistic regression models, this study scanned each biomarker’s potential association with T2D glycaemic deterioration then established causal relationships for some of these identified proteins, such as the Reticulon-4 receptor (NogoR/RTN4R) and the Interleukin-18 receptor 1 (IL18R1).

A limitation of this early study was that no account was taken for potential heterogeneity in disease status (5) which may confound the identification of biomarkers (whose association with disease may vary between sub-clusters). On the other hand, sub-clusters based on a limited number of clinical variables alone may be somewhat “artificial” or subjective in terms of molecular aetiology. Indeed, others (6, 7) have proposed that positioning individuals within a multi-dimensional continuum of biomarkers that globally reflect underlying disease progression or pathology may provide a useful prognostic strategy and better health management.

In the present study, we address these limitations by deploying an unsupervised, bottom-up approach for T2D individual allocation without any pre-assumptions based on clinical characteristics. Thus, we assess the relationships with disease progression and the circulating levels of a large number of molecules belonging to two classes, lipids and proteins.

We have interrogated data from two independent European T2D cohorts, DCS and GoDARTS, within the RHAPSODY consortium (8). T2D Patients with complete lipidomics (180 circulating lipids) and proteomics (1195 circulating proteins) data were queried and analysed through a non-disclosive federated infrastructure (9) using Similarity Network Fusion (SNF) (10). SNF combines different data modalities from the same patients into a similarity network, enabling patient clustering and subsequent feature extraction. This approach reveals the existence of two sub-clusters of individuals in each cohort, with distinct biomarker profiles and clinical characteristics.

## 2 Methods

### 2.1 Study populations

We used data from two type 2 diabetes cohorts: Hoorn Diabetes Care System (DCS) and Genetics of Diabetes Audit and Research in Tayside Scotland (GoDARTS) within the RHAPSODY consortium. RHAPSODY (Risk Assessment and Progression of Diabetes, https://imi-rhapsody.eu) is an Innovative Medicine Initiative project with the goal of enhancing the categorization of individuals with type 2 diabetes and aiding the adoption of innovative approaches for diabetes prevention and treatment.

The DCS cohort recruits almost all T2D patients from 103 GPs in the West-Friesland region of the Netherlands. This prospective, regional cohort study started in 1998 and by 2017, held 12,673 T2D patients with a median of 0.7 years (IQR 0.2-3.7) after diagnosis (11). The study was approved by the Ethical Review Committee of the Vrije Universiteit University Medical Center, Amsterdam. Measurements were labelled anonymously. All laboratory measurements were done on samples taken in a fasted state.

Between 1996 and 2015, GoDARTS recruited 10149 type 2 diabetes patients from the Tayside region of Scotland. Patients in the GoDARTS cohort were not necessarily recruited at the time of diagnosis (12). The GoDARTS study was approved by the Tayside Medical Ethics Committee. All laboratory measurements were measured in a non-fasted state.

Lipidomics and peptidomics are available for a subset of T2D patients in both DCS and GoDARTS cohorts. These were selected with a blood sample close to diagnosis and median diabetes duration of 2.6 and 1.4 years respectively. These data were collected at baseline and generated as part of the RHAPSODY project (4). Of note, individuals were selected without taking into consideration pre-cluster assignments.

Further information on the cohort characteristics can be found in Slieker et al., 2021 (13).

### 2.2 Measurements

Informed consent was obtained from all participants. In DCS, Haemoglobin A1c was measured based on the turbidimetric inhibition immunoassay for haemolysed whole EDTA blood (Cobas c501, Roche Diagnostics, Mannheim, Germany). The levels of triglycerides, total cholesterol and HDL cholesterol were measured enzymatically (Cobas c501, Roche Diagnostics) (11, 12). In DCS and GoDARTS, C-peptide was measured on a DiaSorin Liaison (DiaSorin, Saluggia, Italy). Plasma lipids were determined using a QExactive mass spectrometer (Thermo Scientific), without assessment of the liproprotein particles from which lipids were derived. Plasma protein levels were measured on the SomaLogic SOMAscan platform (Boulder, Colorado, USA). More details on plasma lipidomics and proteomics measurements can be found in Slieker et al., 2023 (4).

### 2.3 Network-based clustering of -omics data

A federated database of T2D cohorts including DCS and GoDARTS has previously been set up as part of the RHAPSODY project. This system enables statistical and machine learning analysis to be performed on cohort data remotely without any disclosure of sensitive data (14–17). The federated database system was interrogated using the R statistical programming language (version 4.0.4). In both DCS and GoDARTS cohorts, patients with complete lipidomics and peptidomics were used for clustering and subsequent statistical analysis. Lipidomics and peptidomics values were centred to a mean value of 0 and a standard deviation of 1 in each cohort using the *dssScale* function in R (*dsSwissKnifeClient* package) (18). Euclidean distances between each pair of patients were then calculated based on the normalized lipidomics or peptidomics data by using *dist2* function from *dssSNF* (*dsSwissKnifeClient* package) (18). *dssSNF* is a wrapper function for *SNFtool* enabling the analysis to be performed on a remote server without sensitive data disclosure. Patient similarity matrices were generated from the Euclidean distance matrices using the *affinityMatrix* function with parameters of K (the number of nearest neighbours) equal to 20 and hyperparameter alpha equal to 5 using *dssSNF*. The patient similarity matrices were then fused using the *SNF* function of the *SNFtool* package (10) with T (number of iterations) equal to 20 using *dssSNF* and clustering was performed using the *spectralclustering* function of the *SNFtool* of *dsSwissKnifeClient* packages (10, 18) Silhouette widths of fused cluster patients were calculated from the similarity matrices using the *Silhouette_SimilarityMatrix* function (*CancerSubtypes* package) (19) to determine the optimal cluster number.

SNF models were validated by bootstrapping tests (n=1000 iterations) that compared to models using randomized Euclidean distance matrices by *randomizeMatrix* (*picante* package) (20). For each simulated model, the same parameters were used, and the number of clusters is set to correspond to the number of clusters in the study model. The significance of each cluster (P-value <=0.05) was calculated by assessing the frequency of achieving a model with an equal or greater mean local cluster coefficient for randomized data. Local cluster coefficient calculation was performed with unweighted, undirected adjacency matrices using the *transitivity* function in the *igraph* package (21). The adjacency matrices were generated from the similarity matrices with the top 2.5% similarity values set as 1 and the rest as 0.

Multi-block Common Dimensions analysis (ComDim) was performed using *federateComDim* from the dsMO package in R (v0.1.12; https://github.com/vanduttran/dsMO) using default parameters. ComDim reduces the lipidomics and proteomics data into a smaller number of dimensions with global and block components for each patient.

### 2.4 Statistical analysis

Logistic regression model was performed using the *ds.glm* function of the *dsBaseClient* (16) package to test differences between clusters for each molecule and clinical measurements. The cluster classification was treated as a dependent categorical variable with age, sex and BMI acting as covariates. Subsequently, for lipids, peptides and clinical measurements, each cluster was subset for the strongly associated (P-value <=0.05) features and the mean values of each group of features in each cluster were calculated. Mean values were used since, to protect the patient’s identity, individual-level data cannot be downloaded from the federated database system. Several mean values for each feature from 5 or more patients were calculated based on the default patients’ order in the remote server for each cluster. The feature values were normalized. Hierarchical clustering was then performed, and the results were visualized as heatmaps using the R package *gplots* (22).

Cox regression was performed using the *dssCoxph* function in the *dssSwissKnifeClient* package. Time to insulin requirement was defined as the length of time from diagnosis until an individual started insulin treatment for a period of more than six months, or alternatively as more than two independent HbA1c measurements greater than 69 mmol/mol (8.5%) at least three months apart and when ≥2 non-insulin glucose-lowering drugs were taken. From the diagnosis until the primary endpoint, no known glucose lowering agents were taken during the monitoring period. Hazard ratios for time to insulin treatment requirement within the clusters were calculated using Cox regression with age, sex and BMI as covariates.

Analyses were performed using the R statistical programming language (version 4.0.4) and Python (Pycharm 2022.2.1). The Benjamini-Hochberg procedure was used to determine the false discovery rate correcting for multiple tests. Figures were produced using *gplots* (version 3.1.1), *ggplot2* (version 3.3.4), *igraph* (version 1.2.6), *circlize* (version 0.4.15), *seaborn* (version 0.12.1), and *matplotlib* (version 3.6.0). The graphical abstract was generated using Biorender.

### 2.5 Data availability statement

Started in 2016, within the RHAPSODY project, we federated 12 patient cohorts totalling over 68000 diabetes patients. We have collected and generated “multi-omics” and genetic data alongside clinical data. All the data have been harmonized and stored in a federated infrastructure to enable remote query and analysis. Data analysis through the federated database has been made possible through the R programming environment with federated analysis packages. Information about accessing the Rhapsody federated database can be found at: https://imi-rhapsody.eu/.

## 3 Results

### 3.1 Multi-omics analysis separates T2D patients into two subgroups

The initial question we wanted to ask was whether T2D patients could be robustly separated based on their combined plasma lipidomics/proteomics signatures and whether molecular differences could highlight novel circulating biomarkers associated with disease severity or progression. To do this, we used a data-driven network-based clustering strategy (SNF) on plasma lipidomics and peptidomics data from a total of 1,134 patients with T2D in two independent cohorts, for which baseline characteristics are given in **Table 1**. A total of 589 (DCS) and 545 (GoDARTS) patients with T2D, for whom complete plasma lipidomics and peptidomics datasets were available, were used for the analysis. The characteristics of the two cohorts were comparable in terms of average age and BMI, with a majority of males present in each case (13). Individual assignments were performed based on SNF for subgrouping similar multi-omics T2D patients.

**Table 1:** Characteristics of the individuals included from the two cohorts.

	DCS	GoDARTS
n	589	545
Male,%	56.7	59.3
Age, years	62 (55.3-69)	61 (53-69)
BMI, kg/m2	29.7 (26.9-33.1)	30.5 (27.2-35.0)
HbA1c, mmol/mol	47.5 (43.5-52.0)	53.0 (46.0-61.0)
C-peptide, nmol/l	1.1 (0.8-1.4)	2.0 (1.4-2.7)
HDL-cholesterol, mmol/l	1.2 (1.0-1.4)	1.3 (1.1-1.5)
LDL-cholesterol, mmol/l	2.5 (2.0-3.0)	2.1 (1.6-2.7)
Triacylglycerol, mmol/l	1.6 (1.2-2.2)	2.0 (1.4-2.7)

Data are displayed as median (IQR), except where otherwise indicated.

**Figure 1:**
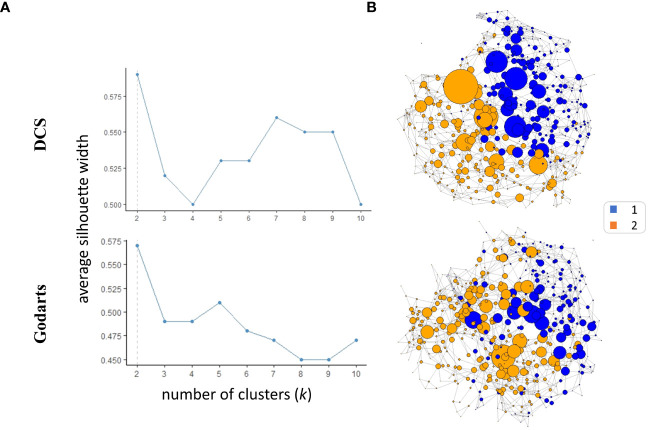
Similarity Network Fusion (SNF) identifies two multiomics clusters in independent cohorts. **(A)** Average silhouette width was calculated for SNF clusters identified in both cohorts with the number of clusters (*k*) ranging from 2 to 10. A high mean silhouette width for a cluster indicates that it is well separated from other clusters and is a measure of clustering quality. **(B)** The similarity networks were generated based on unweighted adjacency matrices. The unweighted adjacency matrices were derived from similarity matrices. In the context of similarity matrices, values exceeding the 97.5th percentile threshold were assigned a value of 1, while values below this threshold were assigned a value of 0.The nodes represent the patients where the size represents the node *betweenness centrality*. Nodes were coloured based on their cluster assignments.

SNF analysis revealed that patients from both GoDARTS and DCS cohorts could be separated into two clusters based on their circulating plasma lipidomics and proteomics data (**Figure 1A**). The significance of the clusters (from now on referred to as subgroups) was validated by bootstrapping (n=1000 iterations) against simulated data (**Supplementary Figure 2**). We also performed the same clustering analysis using single -omics datasets for comparison and showed that the clustering results from the combined (proteomics+lipidomics) dataset are improved compared to single -omics results (**Supplementary Figure 1**). Subgroup 1 comprised 46.5% and 40.7%, respectively, of the cohort patients while subgroup 2 comprised 53.5% and 59.3%, respectively. The similarity between patient subgroups in DCS and GODARTS cohorts is represented as a network where the nodes represent patients and the connections between them (edges) represent the similarity between them (**Figure 1B**). Node connectivity was measured by betweenness centrality, and is reflected by the size of the nodes in the figure. Patients are coloured according to their subgroup labels showing that patient subgroups appear separated from each other in the network. The nodes with high betweenness are mostly positioned in the centre of the network, indicating similarity of these nodes to both subgroups.

To investigate whether similar data-driven separation of patients could be observed using an independent method, we used an unsupervised data fusion approach, *ComDim (CCSWA or Common Dimensions)*, to project patient similarity based on combined lipidomics and proteomics data into two dimensional space (23, 24). For both DCS and GoDARTS cohorts, this analysis showed clear separation of the two patient subgroups identified by SNF along dimension 2. Thus, using an independent method without any *a priori*, we were able to confirm that the subgroups reflect individuals with different underlying plasma lipidomics and proteomics profiles. Details of the features most associated with each common dimension are shown in **Supplementary Table 1**.

### 3.2 Clinical differences between subgroups

**Figure 2:**
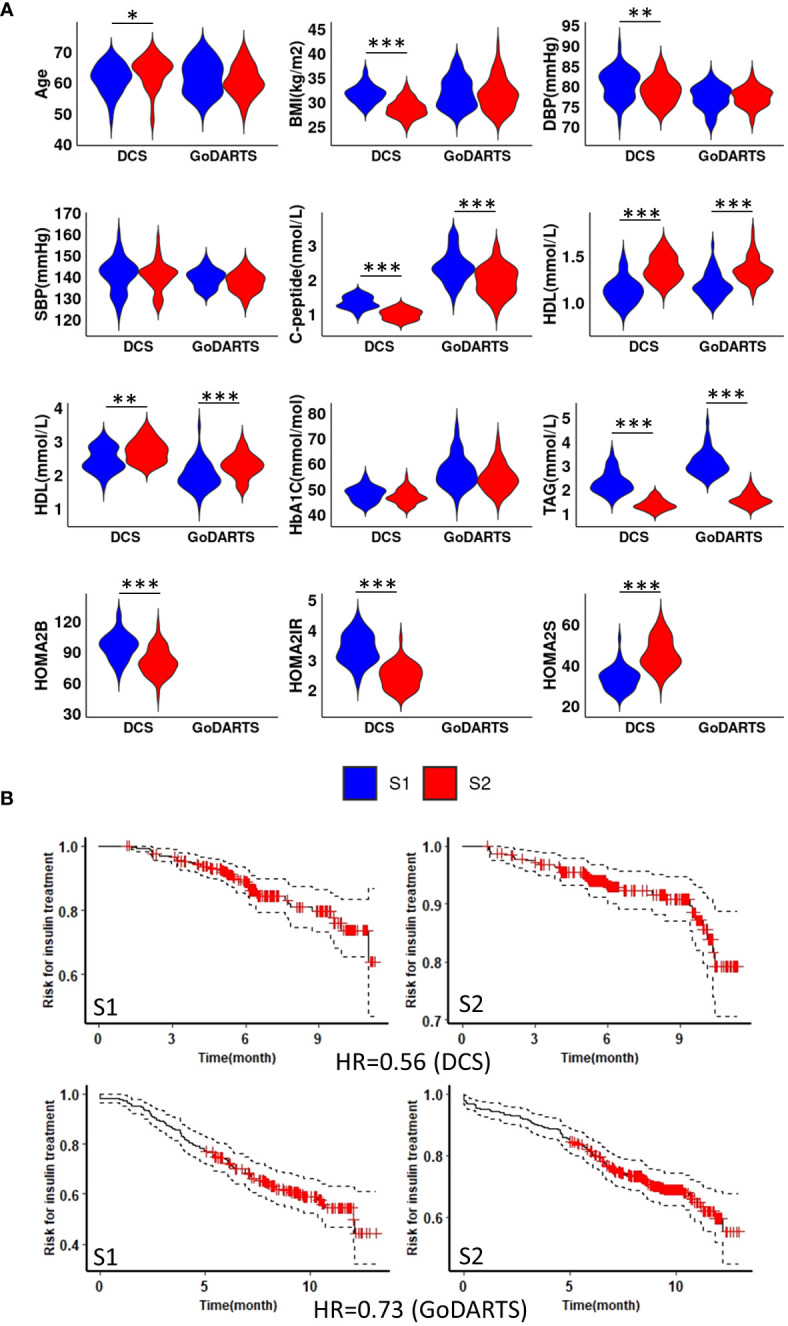
Subgroup 1 shows clinical characteristics of more severe disease and faster glycaemic deterioration. **(A)** Distributions of nine clinical measurements at baseline in both DCS and GoDARTS cohorts for each subgroup. **(B)** Cox proportional hazards models with the time required for insulin treatment between subgroup 1 and subgroup 2 in both DCS (upper) and GoDARTS (lower). The hazard ratio for the second group relative to the first group is shown. DBP, Diastolic Blood Pressure; SBP, Systolic Blood Pressure; BMI, Body Mass Index; HDL, high-density lipoprotein cholesterol; LDL, low-density lipoprotein cholesterol; TAG, Triglycerides; HbA1C, Haemoglobin A1C; HOMA2, Homeostasis Model Assessment 2; DCS, Hoorn Diabetes Care System; GoDARTS, Genetics of Diabetes Audit and Research in Tayside Scotland; S1, Multi-omics subgroup 1; S2, Multi-omics subgroup 2. *P-value<=0.05. **P-value<=0.01. ***P-value<=0.001.

We next sought to determine whether the observed subgroups may differ in terms of their clinical characteristics. Homeostatic model assessment 2 (HOMA2) is a non-invasive, commonly used mathematical model for estimating beta-cell function (-B), insulin sensitivity (-S), and insulin resistance (-IR) (25). In DCS, subgroup 1 and subgroup 2 show significant differences in HOMA2 (*p*=0.0008;4.2e-11;1.1e-09, -B, -IR and -S) and C-peptide (*p*=3.7e-11) level. Patients in subgroup 1 showed higher HOMA2-B, HOMA2-IR and C-peptide levels compared to subgroup 2 (**Figure 2A**). Similar results were observed in GoDARTS (C-peptide *p*=2.5e-06) (**Figure 2A**). Since measurements in subjects from the GoDARTS cohort were performed in a non-fasting state, HOMA2 was not possible for this cohort. Furthermore, glycaemic deterioration, measured as the length of time to start insulin treatment (26), was higher for patients in subgroup 1 than subgroup 2 (Hazard Ratio=0.56; 0.73, DCS; GoDARTS, **Figure 2B**) in each cohort. In order to see how the subgroups compared to previously reported clinically derived T2D clusters in DCS and GoDARTS (5, 13), we assigned each patient to the relevant cluster label and calculated the significance of patient overlap with each molecular subgroup (**Supplementary Table 2**; **Supplementary Figure 4**). In DCS, a small but significant overlap was observed between subgroup 1 and severe insulin-resistant diabetes (SIRD) in DCS (intersect = 91; p-value = 1.13E-6) and mild obesity-related diabetes (MOD) (intersect = 67, p-value = 1.6E-03); and between subgroup 2 and mild diabetes with high HDL-cholesterol (MDH) (intersect = 100; p-value = 7.1E-09). Although less pronounced, a similar trend could also be observed in GoDARTS between subgroup 1 and SIRD (intersect = 51; p-value = 0.085), MOD (intersect = 61; p-value = 0.007) and between subgroup2 and MDH (intersect = 85; p-value = 0.003).

### 3.3 Patient subgroups display distinct molecular features

**Figure 3:**
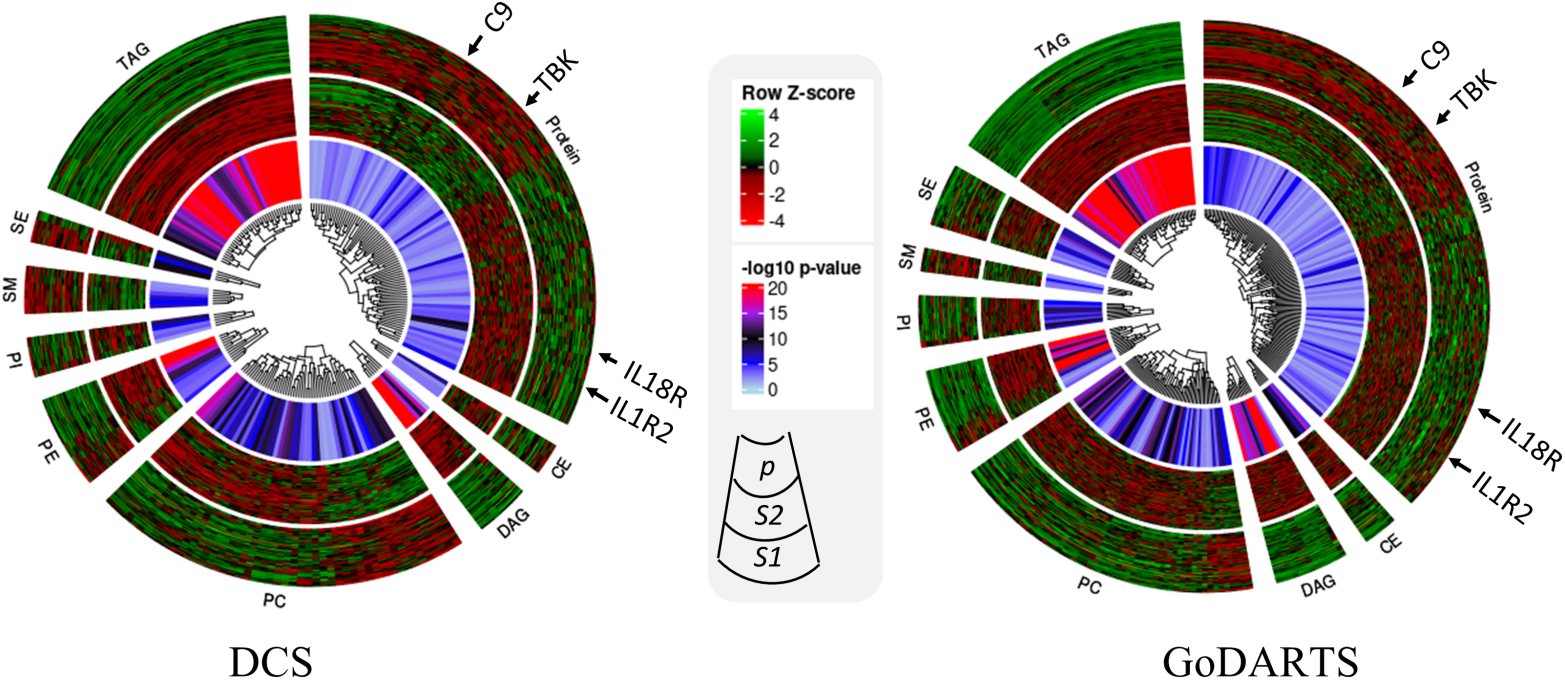
Circular heatmap showing similarities between multi-omics signatures in DCS and GoDARTS. Concentrations of each biomarker (logistic regression; *p*<0.05) were first converted to log(concentration) and then normalised (Row Z-score). Each vertical line in the circular heatmap represents one biomarker and each circle represents one patient. The heatmap was grouped based on first type of molecule, followed by hierarchical clustering. The different layers in the circle are (from outer to inner circle): subgroup1 molecular expression heatmap; subgroup 2 molecular expression heatmap; logistic regression *p*-value. For more details of each individual molecule profile, please refer to **Supplementary Figure 5**. CE, Ceramide; DAG, Diacylglycerols; PC, Phosphatidylcholines; PE, Phosphatidylethanolamine; PI, Phosphatidylinositols; SM, Sphingomyelins; SE, Sterol Esters; TAG, Triacylglycerols; DCS, Hoorn Diabetes Care System; GoDARTS, Genetics of Diabetes Audit and Research in Tayside Scotland; S1, Multi-omics subgroup 1; S2, Multi-omics subgroup 2.

We next investigated the subgroups’ molecular profiles using logistic regression models adjusting for sex, BMI and age. In DCS, 123 proteins and 137 lipids showed significant differences between subgroups. In GoDARTS, 130 proteins and 149 lipids showed significant differences between subgroups (**Figure 3**). 50 significant common peptides and 109 significant common lipids are shared between the DCS and GoDARTS cohorts.

A hierarchical clustering of the multi-dimensional subgroup-associated features is shown as a heatmap and box plot in DCS and GoDARTS (**Figure 3**; **Supplementary Figure 5**). In DCS and GoDARTS, several discriminative features can be observed. For lipids, triacylglycerol (TAG), Diacylglycerol (DAG) and Phosphatidylcholine (PC) show marked differences between clusters in both cohorts.

Patients in subgroup 1 also showed high levels of pro-inflammatory proteins such as interleukin 18 receptor 1 (IL18-R1), interleukin 1 receptor 1 (IL1-R1) and interleukin 19. Moreover, patients in subgroup 1 also had higher levels of proteins related to cellular growth such as growth factor receptor binding protein 2, growth hormone receptor, as well as glucose and fatty acid metabolism-related proteins such as glucose 6 phosphate isomerase (GPI) and 3 hydroxyacyl CoA dehydrogenase (HADH). In contrast, patients in subgroup 2 showed higher levels of immune regulatory proteins such as coactosin-like protein, COD29 antigen and lymphocyte antigen. Furthermore, tyrosine protein kinases family, -Lyn, Fyn and -BTK are also more abundant in subgroup 2 than subgroup 1. Interestingly, several blood coagulation and platelet proteins such as complement component C9, E-selectin and platelet factor 4 showed significant differences between subgroups in both cohorts (**Figure 3**; **Supplementary Figure 5**).

## 4 Discussion

Building on our recent study, which primarily centred on testing individual biomarkers for association with glycaemic deterioration (4), in the present report, we employed an alternative multimodal, multivariate strategy to capture the complexity of molecular interactions associated with T2D. Using an unsupervised network-based data fusion method, SNF, we were able to group T2D individuals with similar multi-omics profiles into two robust subgroups from two independent European cohorts. In the DCS cohort, patients assigned to subgroup 1 and 2 showed clear differences in insulin resistance and beta-cell function as measured by HOMA2-B, -IR, C-peptide, and triglyceride levels, with similar results obtained in the GoDARTS cohort (27). Thus subgroup 1 appears to represent individuals with both increased insulin resistance *and* islet beta cell function at diagnosis.

We also provide evidence from Cox proportional hazards modelling in both cohorts that patients assigned to the different subgroups show altered rates of disease progression as defined by time of diagnosis to requirement for insulin treatment. We note that in the current and prior studies (4), we ascertained the time to insulin initiation based on two criteria: either the duration before an individual commenced insulin treatment post-diagnosis, or when a consistently elevated HbA1c levels was observed despite the use of multiple (>2) non-insulin medications. This is an accepted approach to effectively quantify glycaemic deterioration (26)

Amongst the lipids showing differences between the two subgroups, TAG, DAG and PC showed significantly higher levels in subgroup 1 than in subgroup 2, which potentially reflects an association between circulating fatty acids and insulin resistance (28, 29). In contrast, Sphingomyelin (SM) species were present at higher levels in subgroup 2 compared to subgroup 1. Similar results were also observed in our previous study (4) where decreased levels of sphingomyelin SM 42:2;2 was a predictor of more rapid glycaemic deterioration.

The proteins associated with the present study subgroups fell into a number of different categories, notably immune regulatory related proteins; metabolic enzymes e.g., Alcohol dehydrogenase; hormones and growth factors; signalling proteins; and blood coagulation factors e.g., E-selectin. Notably, several novel T2D biomarkers, which were identified in our previous research as associated with differing rates of T2D progression (4) were significantly altered between subgroups in the present report, suggesting that they form part of a molecular signature associated with disease severity.

Previously, we detected immune regulation proteins IL-18R1, CRELD1 and coactosin-like-protein, to be associated with T2D progression across multiple independent studies (4). Of interest the levels of these proteins are also significantly different between the two subgroups identified in the present study. Moreover, patients in subgroup 1 also showed higher expression levels of other immune-related proteins e.g. CC motif chemokine 15/16/25, ferritin, IL-1R2 and IL-19 and lower level of COD29 and coactosin-like protein (**Supplementary Figure 5**) highlighting the value of using an unsupervised multivariate approach for detecting disease related signatures which may be missed by more conventional supervised approaches.

The goal of the present study was to use plasma lipidomics and proteomics data to uncover -omics signatures related to disease severity. Thus, the two subgroups we identify should be primarily seen as tools to help investigate molecular signatures underlying disease rather than as a means to cluster or stratify patients. Nevertheless, it is meaningful to compare these subgroups with previously identified data driven clusters (5) since these latter clusters have been validated and extensively analysed in the same two cohorts (8, 13). The comparison showed small but significant overlap between subgroup1 and SIRD and MOD, and between subgroup2 and MDH indicating that although there are some similarities, the multi-omics-based subgroups do not closely correspond to any of these previously identified clusters. This underscores the fact that clustering will give different results depending on what data is used and is best employed to observe tendencies, trends or patterns in underlying data rather than for strict patient stratification.

During our analysis, we also tested the subgroups for enrichment of ~400 known genetic loci associated with T2D (30) since this could provide evidence for causal association to disease. However, we found no significant enrichment of any of the T2D variants within the subgroups, possibly reflecting lack of power to detect such associations. The data used in this study, although limited in terms of absolute number of patients, is substantial considering the cost of generating the proteomics data alone (>1000USD/sample). Nevertheless, although high cost precludes performing this type of analysis on large populations or routinely in the clinic, it would be beneficial in future studies to profile large numbers of individuals with T2D with selected panels of protein/lipid biomarkers similar to the ones presented here. Measuring biomarkers over time is especially relevant as T2D is dynamic and underlying molecular signatures may change as the disease progresses. Indeed, biomarkers such as TAG and DAG can be subject to changes reflecting an individual’s lifestyle and/or medication. These aspects will need to be assessed in future prospective studies.

## 5 Limitations of the study

The subgroups in our study were derived primarily from European patients. Consequently, the applicability of these results to other ethnic groups is uncertain. In both DCS and GoDARTS, patients were not necessarily recruited after diagnosis. This heterogeneity in the disease duration could potentially have an effect on the blood measurements obtained. Moreover, as mentioned above, the absence of C-peptide measurements in the fasted state precluded HOMA assessments in the GoDARTS cohort. The effect of fasting or non-fasting state on the multi-omics profiling in the current study is still not fully clear and needs further investigation (31, 32).

## 6 Conclusions

We demonstrate that an unsupervised, “bottom-up” multi-omics approach can segregate T2D patients into 2 subgroups capturing differences in insulin resistance and glycaemic deterioration. Several classes of biomarkers, notably those involved in immune processes, were most strongly associated with these subgroups and future investigations will be necessary to establish their causal roles, if any, in disease progression.

## Data availability statement

The original contributions presented in the study are included in the article/**Supplementary Materials**. Further inquiries can be directed to the corresponding authors.

## Ethics statement

Ethical review and approval was not required for the study on human participants in accordance with the local legislation and institutional requirements. Written informed consent from the patients/participants or patients/participants’ legal guardian/next of kin was not required to participate in this study in accordance with the national legislation and the institutional requirements.

## Author contributions

SL: Formal analysis, Investigation, Methodology, Writing – original draft. ID: Methodology, Software, Data curation, Writing – review & editing. VT: Methodology, Writing – review & editing. CH: Methodology, Investigation, Writing – review & editing. DK: Methodology, Writing – review & editing. MH: Conceptualization, Resources, Writing – review & editing. JB: Conceptualization, Data curation, Writing – review & editing. LH: Conceptualization, Writing – review & editing. RS: Methodology, Writing – review & editing. LD: Data curation, Methodology, Writing – review & editing. MG: Methodology, Writing – review & editing. CK: Methodology, Writing – review & editing. FM: Methodology, Writing – review & editing. KS: Funding acquisition, Methodology, Supervision, Writing – review & editing. PE: Methodology, Writing – review & editing. EP: Funding acquisition, Supervision, Writing – review & editing. GR: Conceptualization, Funding acquisition, Resources, Supervision, Writing – review & editing, Project administration, Writing – original draft. MI: Funding acquisition, Methodology, Resources, Supervision, Writing – review & editing, Writing – original draft.
